# A Novel PTEN Frameshift Variant in a Child With Autism Spectrum Disorder and Macrocephaly: A Case Report

**DOI:** 10.7759/cureus.100834

**Published:** 2026-01-05

**Authors:** Margarida Moreno Fernandes, Mariana Rodrigues Neto, Mariana Sá Pinto, Teresa Pena Fernandes, Ana Catarina Maia, Isabel Ayres Pereira, Armanda Passas, Ana Grangeia, Cristina Madureira

**Affiliations:** 1 Pediatrics, Unidade Local de Saúde Gaia/Espinho, Vila Nova de Gaia, PRT; 2 Genetics, Unidade Local de Saúde Gaia/Espinho, Vila Nova de Gaia, PRT; 3 Genetics, Unidade Local de Saúde São João, Porto, PRT

**Keywords:** autism spectrum disorder (asd), child, macrocephaly, pten, pten hamartoma tumor syndrome (pths)

## Abstract

PTEN hamartoma tumor syndrome (PHTS) is a rare genetic condition associated with neurodevelopmental disorders, macrocephaly, and increased cancer risk. We report the case of a four-year-old girl with congenital hypothyroidism, progressive macrocephaly, and global developmental delay, later diagnosed with autism spectrum disorder (ASD). Brain MRI revealed megalencephaly with prominent extra-axial spaces and a diffusely thickened corpus callosum. Genetic testing identified a novel frameshift variant in the *PTEN* gene. This case highlights clinical findings that should raise suspicion for PHTS and was documented to emphasize the importance of recognizing *PTEN*-related disorders in children presenting with autism and macrocephaly, particularly when oncologic manifestations are not yet evident, thereby supporting early genetic diagnosis and appropriate surveillance.

## Introduction

PTEN hamartoma tumor syndrome (PHTS) encompasses a clinical spectrum of related allelic disorders characterized by marked phenotypic heterogeneity. Its manifestations commonly include macrocephaly, autism spectrum disorder (ASD), and developmental delay, reflecting the central role of *PTEN* in neurodevelopment and growth regulation [1-3]. ASD is a neurodevelopmental condition defined by impairments in social communication and interaction, together with restricted and repetitive patterns of behavior [4]. Macrocephaly is reported in 14-34% of individuals with ASD, and pathogenic *PTEN* variants are identified in approximately 17-20% of children who present with both ASD and macrocephaly [5]. Furthermore, developmental delay associated with macrocephaly, even in the absence of other classic PHTS features, has been linked to *PTEN* pathogenic variants in 1-8% of affected patients.

In addition to neurodevelopmental manifestations, PHTS is associated with an increased lifetime risk of several malignancies, including breast, thyroid, renal, endometrial, and colorectal cancers, underscoring the importance of early diagnosis for appropriate surveillance and long-term management. Given the multisystemic nature of PHTS, long-term follow-up is essential to monitor oncologic and systemic risks, as early detection of complications significantly improves outcomes [6].

This case highlights clinical findings that should raise suspicion for PHTS and was documented to emphasize the importance of recognizing *PTEN*-related disorders in children presenting with autism and macrocephaly, particularly when oncologic manifestations are not yet evident, thereby supporting early genetic diagnosis and appropriate surveillance.

## Case presentation

Developmental and behavioral profile

We present a four-year-old girl with congenital hypothyroidism diagnosed through newborn screening and treated with levothyroxine from the first month of life, with consistently well-controlled thyroid function. At 24 months, her family raised concerns regarding delayed development, reduced social interaction, and snoring without clear apneas during sleep.

Clinically, she demonstrated poor verbal communication (two-word vocabulary without phrase construction), inconsistent eye contact, no response to her name or simple commands, absence of protoimperative or protodeclarative pointing, and lack of symbolic play. Social interaction was atypical, with restricted interests, repetitive behaviors, instrumental use of the adult’s hand, marked sensory hypersensitivity, frequent spinning movements, and motor and verbal stereotypies.

Developmental assessment with the Schedule of Growing Skills II (SGS II) at 24 months showed significant delay (≥2 SD) in visual skills and receptive and expressive language [7]. The Modified Checklist for Autism in Toddlers (M-CHAT™, © 2009 Robins, Fein, & Barton) was positive (eight failed items, three critical) [8]. At 36 months, the Griffiths III scale confirmed global developmental delay [9]. The diagnosis of ASD was further supported by a certified neurodevelopmental team using the Autism Diagnostic Interview-Revised (ADI®-R) and Autism Diagnostic Observation Schedule, Second Edition (ADOS®-2) [10,11].

Physical findings, growth parameters, and laboratory results

**Figure 1:**
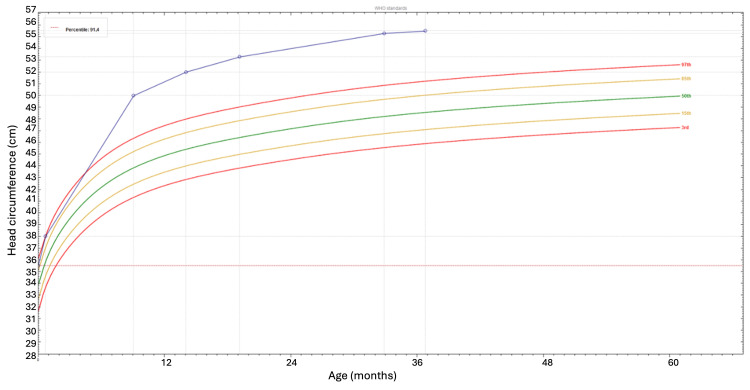
Evolution of head circumference over time, showing values between the 85th and 97th percentiles until the first month of life and persistently above the 97th percentile thereafter, according to World Health Organization (WHO) growth curves. Based on the World Health Organization (WHO) growth curves [12].

Her head circumference was between the 85th-97th percentile at birth, reached the 97th percentile at one month, and remained above the 97th percentile thereafter (Figure 1). Physical examination revealed macrocephaly, mouth breathing, tonsillar hypertrophy, and pale inferior turbinates. Neurological examination was unremarkable.

Family history included asthma in the mother and a maternal half-sibling, with no reported neurodevelopmental, psychiatric, epileptic, or oncologic conditions. No family members, including both parents, presented clinical features suggestive of PHTS, such as mucocutaneous lesions or hamartomas.

Blood tests were normal aside from congenital hypothyroidism that was controlled since the first month of life with levothyroxine. 

Neuroimaging and genetic findings

**Figure 2:**
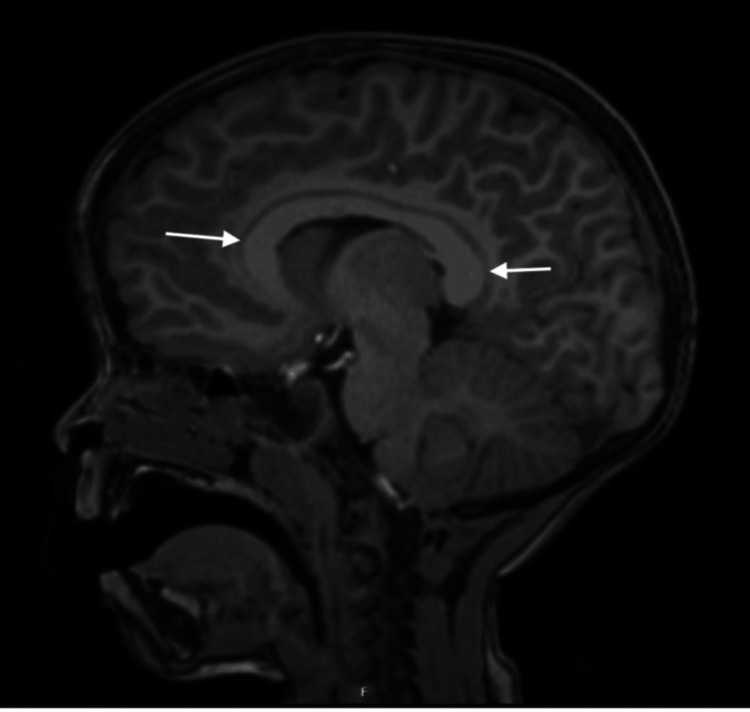
Sagittal T1-weighted brain MRI showing a diffusely thickened corpus callosum (arrows). The corpus callosum appears globally enlarged, a feature described in *PTEN*-related megalencephaly.

Brain MRI with spectroscopy showed megalencephaly with a frontal hump, increased extra-axial spaces, proportional enlargement of encephalic volume, thickening of the corpus callosum (>97th percentile), and nonspecific prominent perivascular spaces (Figure 2). Thyroid ultrasound revealed a small right-lobe remnant and an absent left lobe.

Genetic investigations included a normal Fragile X study and a normal array CGH. *PTEN* sequencing identified a heterozygous likely pathogenic frameshift variant, NM_000314.8:c.302del p.(Ile101Thrfs*12), not previously reported. The premature stop codon is predicted to result in nonsense-mediated decay, consistent with PHTS.

Follow-up and multidisciplinary care

Given the autosomal dominant inheritance and 50% recurrence risk, the patient and her parents were referred for genetic counseling. Parental genetic testing was not available at the time of submission; thus, the possibility of a de novo variant could not be excluded.

Ophthalmologic evaluation was normal. ENT evaluation demonstrated conductive hearing loss with a type B tympanogram. Due to persistent snoring and suspected obstructive sleep apnea unresponsive to medical treatment, she underwent tonsillectomy, myringotomy, and tympanostomy tube placement.

She continues follow-up with endocrinology and early intervention services, including speech therapy, occupational therapy with sensory integration, and child psychiatry follow-up for behavioral comorbidities.

## Discussion

*PTEN*, located on chromosome 10q23, encodes a phosphatase regulating the PI3K-AKT-mTOR pathway involved in neurogenesis, synaptic development, neuronal migration, and cell-growth control [13,14]. Loss-of-function variants underlie the multisystemic manifestations of PHTS.

A significant proportion of children with ASD and macrocephaly carry *PTEN* pathogenic variants [5], and 25-50% of individuals with *PTEN* variants may meet ASD criteria [2,15,6,16]. PHTS may present with hamartomas, macrocephaly, developmental delay, epilepsy, vascular malformations, thyroid disease, gastrointestinal polyps, immune dysregulation, and increased risk of malignancies such as breast, thyroid, renal, colorectal, and endometrial cancer, and melanoma [17].

International guidelines recommend *PTEN* sequencing when macrocephaly is associated with ASD or developmental delay [6]. Pediatric scoring tools incorporate features such as macrocephaly, neurodevelopmental findings, dermatologic/vascular lesions, and tumor risk [6,17,18].

Early identification is critical to initiate cancer surveillance, including thyroid palpation and ultrasound during childhood [6,17]. Recommended surveillance for PHTS is shown in Table 1.

**Table 1:** Recommended screening for patients with PHTS. Surveillance recommendations for PTEN hamartoma tumor syndrome (PHTS) are adapted from published guidelines [6,17,18].

Screening	Timing
Neck palpation and thyroid ultrasound	At diagnosis and from 7 years old, annually or from 12 years old; if no nodules: repeat every 3 years; if nodules are detected: general guidelines for thyroid nodules; consider following more closely if risk (history of chemotherapy or radiation)
Breast self-exam	From 18 years old
Clinical breast exam	From 25 years old, every 6–12 months
Breast imaging (mammogram or MRI with contrast)	From 30–35 years old, annually
Renal ultrasound	Every 1–2 years
Renal CT/MRI	If abnormalities are seen on ultrasound
Endometrial biopsy	From 45 years old, every 1–2 years
Transvaginal ultrasound	In postmenopausal women, as needed
Colonoscopy	From 35 years old, every 5 years or more frequently if symptoms/polyps
Skin exam	From 18 years, annually
Brain MRI	If suspicious symptoms (persistent headache, focal signs)
Screening based on family history	5–10 years before the earliest cancer in the family

Children with *PTEN*-related neurodevelopmental disorders benefit from a comprehensive assessment of cognitive, language, attentional, motor, and adaptive domains [4,19].

Management is multidisciplinary. mTOR-modulating therapies such as sirolimus have shown potential benefit in isolated pediatric cases but remain investigational [17].

In pediatric patients, the combination of progressive macrocephaly and neurodevelopmental disorders, particularly ASD, should be considered a red flag for underlying genetic conditions such as PHTS. As oncologic manifestations often arise later in life, early neurodevelopmental presentations may represent the first opportunity for diagnosis. Timely identification of *PTEN* pathogenic variants enables appropriate cancer surveillance, multidisciplinary follow-up, and informed genetic counseling, even in the absence of tumor-related features at presentation.

This case illustrates how early neurodevelopmental features and progressive macrocephaly can be the initial manifestations of PHTS, preceding oncologic findings and allowing timely implementation of surveillance strategies.

## Conclusions

PHTS represents a broad clinical spectrum in which macrocephaly, developmental delay, and ASD may be early or isolated presenting features. This case reinforces the importance of *PTEN* sequencing when suspicion arises, as early diagnosis guides surveillance and informs genetic counseling. Identification of a previously undescribed *PTEN* variant expands the understanding of PHTS genotype-phenotype correlations.
